# Individual Protective Covers Improve Yield and Quality of Citrus Fruit under Endemic Huanglongbing

**DOI:** 10.3390/plants13162284

**Published:** 2024-08-17

**Authors:** Susmita Gaire, Ute Albrecht, Ozgur Batuman, Mongi Zekri, Fernando Alferez

**Affiliations:** 1 Southwest Florida Research and Education Center, Institute of Food and Agricultural Sciences (UF-IFAS), University of Florida, Immokalee, FL 34142, USA; sgaire@ncsu.edu (S.G.); obatuman@ufl.edu (O.B.); 2 Hendry County Extension, Institute of Food and Agricultural Sciences (UF-IFAS), University of Florida, 1085 Pratt Blvd, LaBelle, FL 27695, USA; maz@ufl.edu

**Keywords:** citrus greening, *Candidatus Liberibacter asiaticus*, citrus fruit quality, protective netting, psyllids

## Abstract

The use of individual protective covers (IPCs) to protect newly planted citrus trees from Huanglongbing (HLB) infection is being widely adopted in Florida, an HLB-endemic citrus-producing area. It is known that IPCs positively influence most horticultural traits, increasing tree growth, flush expansion, and leaf size, enabling trees to sustain balanced carbohydrate metabolism by preventing *Candidatus Liberibacter asiaticus* (*C*Las) infection, and inducing higher leaf chlorophyll levels. This may result in more productive trees. However, as the tree grows, IPCs eventually are removed, typically between 2 and 3 years after their initial installation. Once IPCs are removed, trees become exposed to the Asian citrus psyllid (ACPs) and ultimately become infected. In this work, we covered Valencia sweet orange trees with IPCs for 30 months, until the trees entered fruit-bearing age. We investigated how the IPC protection of newly planted trees for 30 months influenced the fruit quality and yield of “Valencia” trees for three consecutive seasons after IPC removal compared to non-covered trees. The use of IPCs kick-started the newly planted citrus trees, resulting in higher yields and fruits with better internal and external quality. After 30 months of IPC protection, tree canopies were larger and denser, supporting more fruit per tree than non-protected trees for three consecutive seasons, even though by the end of the first season after IPC removal, the trees were HLB-positive. Tree height, scion diameter, canopy volume, and leaf area were significantly improved compared to non-covered trees. Additionally, fruit quality was significantly improved in the three seasons following IPC removal compared to non-covered trees. However, a decline in quality was measurable in fruit from IPC trees after the second harvesting season, with trees affected by HLB. Based on the results from this study, we conclude that the benefits from IPC protection may last for at least three consecutive seasons once trees enter the productive age, despite *C*Las infection within 12 months after IPC removal.

## 1. Introduction

Huanglongbing (HLB or citrus greening), the destructive disease that has decimated the citrus industry in Florida, USA, is associated with the phloem-limited bacterium *Candidatus Liberibacter asiaticus* (*C*Las) and is now widespread and endemic in the state [1]. Despite the endemic nature of HLB in Florida, citrus growers continue to plant new trees. Controlling HLB and the spread of the vector, the Asian Citrus Psyllid (ACP, *Diaphorina citri*) is critical, especially in newly planted citrus trees, to ensure survival and productivity in an environment of high ACP pressure and neighboring mature infected trees, allowing rapid spread of the disease. Young citrus trees are more vulnerable to both ACP infestation and *C*Las infection due to more frequent flushing, as psyllids prefer young flushes [1], and protection from the vector psyllid is crucial. Importantly, infection during the early growth stage prevents trees from ever becoming productive [2]. Vector exclusion is an effective strategy for HLB management. Growing citrus under protective screens (CUPS) is effective in excluding psyllids and allows for rapid, healthy growth and the production of high-quality fruit [3,4]. However, due to the high cost, this production system is only viable for fresh fruit production and not feasible for the large-scale production of varieties used for juice processing, such as Valencia or Hamlin (*Citrus sinensis* L. Osbeck), which represent more than 90% of citrus grown in Florida. An alternative method is the use of individual protective covers (IPCs) for young trees. IPCs are polyethylene mesh bags with a pore diameter smaller than the size of a psyllid (≈0.6 mm), preventing the insects from accessing the new flushes and hence impeding pathogen transmission. Both in solid blocks of newly planted trees and as resets of scattered new trees occupying vacant planting spots after the removal of dead or unproductive trees in infected mature groves, this system is being increasingly adopted by citrus growers in Florida. We have shown previously that IPCs are effective in protecting newly planted trees from HLB by excluding the *C*Las-carrying ACP vector [5,6]. In addition, IPCs altered microclimatic conditions in the tree canopy and positively influenced most of the horticultural traits measured, increasing tree growth, flush expansion, and leaf size. At the same time, less leaf drop was observed, and the canopy was denser in trees under IPCs compared to non-covered trees [6]. Finally, IPCs enabled trees to sustain balanced carbohydrate metabolism by preventing *C*Las infection and induced higher leaf chlorophyll levels [7]. Since leaf chlorophyll levels are positively correlated with photosynthesis rate and plant productivity [8], it is expected that IPC-covered trees will be more productive compared to trees without IPCs as they reach maturity and enter fruit-bearing age.

Typically, young trees may be covered with IPCs for up to three years before they are removed, but this depends on the variety, rootstock–scion combination, growth pattern, grove management, and size of the IPCs [5]. After IPC removal, trees become exposed to ACPs and ultimately become infected. The rate of infection in such trees has not been determined to date. At the same time, many varieties that do not need cross-pollination may have begun to bloom and set fruit already. “Valencia” sweet orange (*Citrus sinensis*), which is the most cultivated variety in Florida, is self-fertile and can therefore set fruit in an IPC-enclosed environment. This opens the possibility of setting an earlier-than-usual crop, and accelerating and increasing the return on investment for growers. One main consequence of HLB is the great reduction in fruit yield and quality. HLB-affected fruits have less soluble solids and higher titratable acidity, among other unfavorable qualities resulting from changes in secondary metabolites [9]. This results in juice that is described as distinctly bitter, sour, salty/umami, metallic, musty, and lacking in sweetness and fruity/orange flavor [10]. Fruit yield has been dramatically reduced in recent years, mostly due to extensive preharvest drop, smaller fruit size, and the general decline in HLB-affected trees [11]. It has been determined that in “Valencia” sweet orange, the drop rate of mature fruit is significantly greater for trees exhibiting severe HLB symptoms than for mildly symptomatic trees [12].

Here, we investigated how the IPC protection of newly planted trees for 30 months influenced the fruit quality and yield of “Valencia” trees for three consecutive seasons after IPC removal compared to non-covered trees. We also determined the *C*Las infection rate after IPC removal. We found that the delay in infection provided by the IPCs provided a kick-start advantage to newly planted trees that resulted in significantly higher yields and better internal fruit quality after three years, even though the trees became infected within 12 months after IPC removal.

## 2. Materials and Methods

### 2.1. Plant Material

The experiment was conducted at the University of Florida, Southwest Florida Research and Education Center (SWFREC) research farm in Immokalee, Collier County, FL (26°27′51.4″ N, 81°26′39.9″ W). HLB-free 18-month-old trees composed of a sweet orange “Valencia” (*Citrus sinensis* L. Osbeck) scion grafted on a “Cleopatra” mandarin (*C. reticulata*) rootstock obtained from a commercial citrus nursery (Southern Citrus Nurseries, Dundee, FL, USA) were used for establishing a new plot for the experiment.

### 2.2. Experimental Design

Ninety trees were planted in January 2018 in six rows of 15 trees each at a spacing of 8 feet (2.4 m) within rows and 22 feet (6.7 m) between rows. The trees were arranged in a completely randomized 2 × 3 factorial design with five replications, each consisting of linear plots of 3 trees, as described in a previous work [6]. The first factor (tree cover) had two levels: (1) IPC and (2) no IPC. The second factor (insecticide rate) had three levels: (1) the full recommended rate (Full), (2) half the recommended rate (Half), and (3) no insecticides (Zero), as specified in Gaire et al., 2022 [6]. We, therefore, had total of 6 treatment combinations/interactions.

### 2.3. Treatments and Experiment Timeline

**Figure 1:**
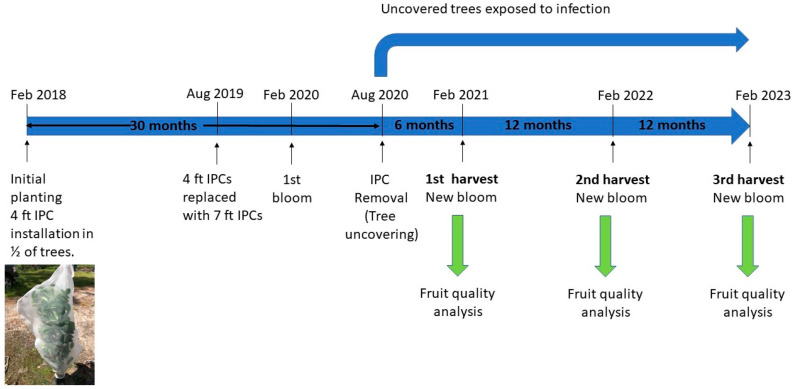
Timeline of experiment. Trees were covered with IPCs or left exposed for 30 months. Both covered and non-covered trees bloomed and set fruit for the first time 24 months after planting (February 2020). In August 2020, trees with IPCs were uncovered, exposing trees to psyllids. In February 2021, first yield and fruit quality assessment were conducted. These assessments continued for two more seasons.

Individual protective covers (IPCs, Tree defender Inc., Dundee, FL, USA) were installed on 45 randomly selected trees at the time of planting (January 2018). The IPCs were composed of monofilament high-density polyethylene (HDPE) with a mesh size of 50 (0.297 mm diameter holes). A PVC pole was installed to support each IPC and the covers were tied with zip ties at the base of the trunk to prevent psyllids and other insects from entering. During the first 18 months, the trees were covered with 4 ft (1.2 m) tall IPCs until the cover restricted canopy expansion. The 4 ft IPCs were then replaced with 7 ft (2.1 m) tall IPCs to allow the canopy to further expand. For these large-sized IPCs, the supporting PVC poles were equipped with metal spreaders to support the net. The 7 ft IPCs remained on the trees for an additional 12 months until removal in August 2020. The timeline of the experiment is depicted in Figure 1.

Insecticide treatments were performed as described before [6]. The insecticides were diluted in water and each tree received a soil drench of 300 mL material per application. The trees were irrigated three times a week by under-tree microjets. Diamond-R 8-8-8 young tree blend (Diamond R Fertilizer, Fort Pierce, FL, USA) was applied at a rate of 227 g per tree in year 1, and 454 g per tree in years 2 and 3. Diamond R CitriBlend 12-8-6 controlled-release fertilizer (Diamond R Fertilizer, Fort Pierce, FL, USA) was applied at a rate of 227 g per tree in year 4. Weeds were uniformly managed in the experimental plot as needed using standard grower practices. Upon IPC removal 30 months after planting, the insecticide treatments and irrigation continued without changes for the remainder of the experiment (30 additional months).

### 2.4. Candidatus Liberibacter Asiaticus (CLas) Detection

Quantitative real-time polymerase chain reaction (qRT-PCR) was conducted for *C*Las detection using a 7500 Fast Real-Time PCR system (Applied Biosystem, Foster City, CA, USA). The trees were sampled for *C*Las detection during spring and summer of 2019, and 2020. Three to four mature leaves from recent flushes were randomly collected from the middle tree of each of the five 3-tree replicated plots. The petioles and midribs of the leaves were excised, minced with a razor blade, lyophilized in a FreeZone 6 freeze-dry system (Labconco, Kansas City, MO, USA), and pulverized using a mini bead beater (Biospec products, Inc., Bartlesville OK, USA). DNA from 100 mg of pulverized leaves was extracted using a Wizard Magnetic 96 DNA Plant System (Promega Corporation, Madison, WI, USA) according to the manufacturer’s instructions. The DNA was quantified using a microplate reader (Synergy HTX Multimode Reader, Biotek instruments, Inc., Winooski, VT, USA) and normalized to 10 ng/µL. The analysis was conducted using primers (HLBas and HLBr), and an HLBp probe. The amplification protocol was 95 °C for 20 s followed by 40 or 50 cycles at 95 °C for 1 s and 58 °C for 40 s [6]. In our experiment, Ct-values of less than 36 were considered to be *C*Las-positive and those that were equal to or greater than 36 were considered to be *C*Las-negative. Samples for which no qPCR product was detected after 40 cycles were assigned a value of 41. IPCs were briefly removed for leaf sample collection and replaced immediately thereafter at each time point.

### 2.5. Tree Size

Tree height, canopy volume, trunk diameter (scion and rootstock), and leaf area index were assessed on every middle tree of each 3-tree plot as previously described [6]. Tree height was measured from the soil surface to the top of the tree using a tape measurer (Komelon, Waukesha, WI, USA) and avoiding any erratic shoots. Canopy diameter was measured parallel and perpendicular to the row, and canopy volume was calculated using the formula by Wutscher and Hill (1995) [13]:

canopy volume = [(diameter parallel to row × diameter perpendicular to row) × height]/4.

The leaf area index (LAI) was determined using a LIA-2200C Plant Canopy Analyzer (LI-COR Biosciences, Lincoln, NE, USA). Tree height, canopy volume, and LAI were measured during summer 2020, 2021, and 2022.

Scion and rootstock diameters were measured at 5 cm above and below the graft union, respectively, during summer (July) of 2020, 2021, and 2022 using a Vernier caliper (Fowler high-precision, Auburndale, MA, USA). Trunk diameters were measured in two perpendicular directions and averaged.

### 2.6. Fruit Quality and Yield

Fruits (four replicates of five fruits) were randomly collected from the 3 IPC-covered trees in each replicated plot of 5 trees in February of every year. Fruit was so scarce in the non-covered trees that all harvested fruits were used for quality determination. Juice was extracted with a hand reamer. Brix was determined by using a table-model refractometer with temperature compensation (Hanna Instruments, Smithfield, RI, USA). The percentage of titratable acidity (TA) was determined by titrating sodium hydroxide to a phenolphthalein endpoint. The results are presented as the Brix-to-acid ratio. Fruit peel color was measured using a portable colorimeter (Konica Minolta, CR-400, Tokyo, Japan). For this, three different readings were obtained along the equatorial circumference of each fruit. The results are presented as the a*/b* color ratio. Yield was assessed in three consecutive years in the second week of February by counting the number of fruits in each 3-tree plot at harvest. Fruit diameter was measured with a digital caliper and fruit was classified as either large (commercially acceptable) when the diameter was >60 mm or small (not commercially acceptable) when the diameter was smaller than 60 mm.

### 2.7. Statistical Analysis

An analysis of variance (ANOVA) was conducted to determine the effect of tree cover and insecticide rate and their interaction. Mean separation was performed by Tukey’s honestly significant difference (HSD) test. Differences were defined as statistically significant when *p* < 0.05.

## 3. Results

### 3.1. CLas Infection

**Figure 2:**
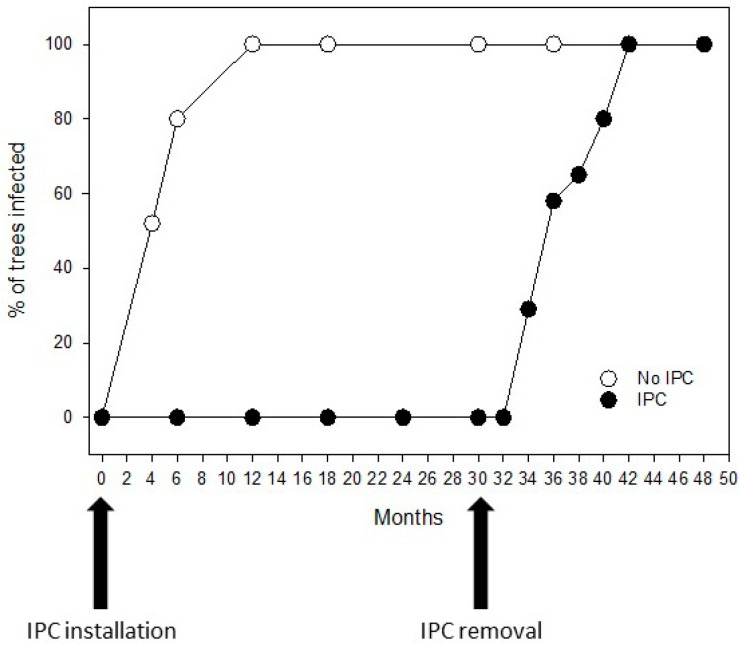
Rate of HLB progression after IPC removal. White symbols represent non-covered trees, showing 80% infection by 6 months and 100% after 12 months. Black symbols represent trees that were covered with IPC for 30 months and remained non-infected during that time. Arrows indicate month of IPC installation and removal.

Trees were covered with IPCs for 30 months. For comparison, trees without covers were also used. Both sets of trees were planted in February 2018. After 30 months, in August 2020, trees covered with IPCs were uncovered and left exposed to natural psyllid infestation for the rest of the experiment. Trees not covered with IPCs in the beginning of experiment started to test positive for *C*Las as early as 4 months after planting, and by 6 months, 80% of the trees were already infected (Figure 2). After 12 months, all non-covered trees tested positive for *C*Las, whereas trees covered with IPCs remained *C*Las-free for 30 months. After IPC removal, infection progressed in these trees at the same rate as in non-covered trees. By month 42, 12 months after IPC removal (August 2022), all trees in the trial were *C*Las-positive (Figure 2). No significant effect of insecticide rate was found on *C*Las infection.

### 3.2. Tree Size

Six months after IPC removal, the effects on tree height were still significant (Table 1). Trees that had been covered with IPCs for 30 months were significantly taller (2.1 m) than non-covered trees (1.6 m). This effect was diminished by the second season. By the last season (spring 2023), 30 months after IPC removal, the differences in tree height were still significant (2.55 m compared to 1.8 m, Table 1). However, no significant effect of insecticide rate was found on tree height. Trunk diameters were also larger in IPC trees compared to non-IPC trees. Rootstock and scion diameter were not significantly affected by the IPC treatment but were significantly increased by insecticide treatment for the scion (Table 1).

Canopy volume was not significantly different between IPC and non-IPC trees 6 months after IPC removal, but after 30 months, the differences were significant (Table 1). In contrast, canopy volume was significantly affected by the insecticide rate at both 6 and 30 months after IPC removal, and trees that had received the full dose of insecticide had a significantly larger volume (1.2 m^3^) than trees that had received no insecticide until IPC removal (0.5 m^3^) by 6 months and by 30 months (1.54 m^3^ as compared to 0.55 m^3^). The leaf area index was significantly larger in trees with IPCs than in and trees without IPCs (2.21 compared to 1.10 by 6 months and 3.7 compared to 1.6 by 30 moths), but there was no significant effect of insecticide rate (Table 1).

### 3.3. Yield and External and Internal Fruit Quality

**Figure 3:**
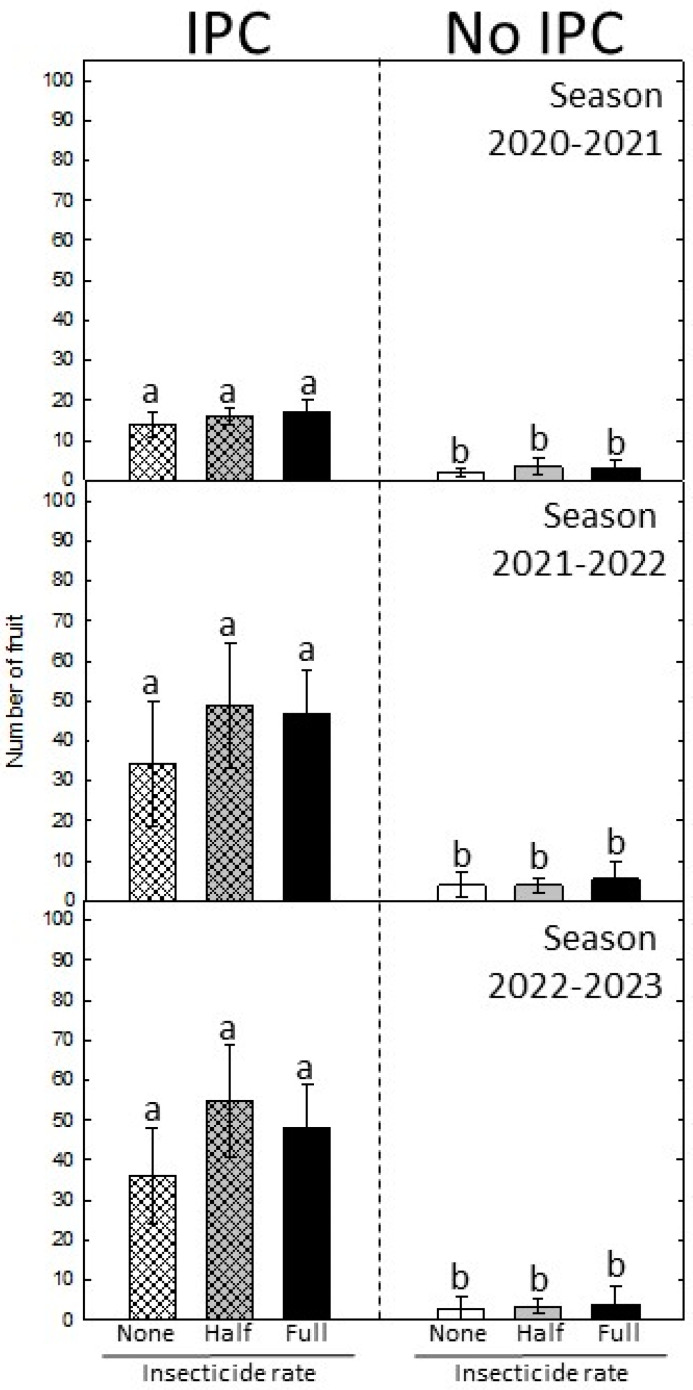
Number of fruits per treatment in every season. Values are means of 5 replications of 3 trees ± SD. Different letters indicate significant differences between IPC and non-IPC trees (*p* < 0.05).

We monitored fruit set and yield in both IPC and non-IPC trees for three consecutive seasons. Trees, irrespective of treatment, bloomed for the first time in February 2020, 24 months after planting. This means that fruit set for the first crop occurred when IPC trees were still covered with the IPCs. In this first season (2021), fruit yield was still low as the trees were young. However, the number of fruits was significantly (*p* < 0.05) higher in IPC trees as compared to non-IPC trees, irrespective of insecticide rate (Figure 3). In the second and third seasons (2022 and 2023, respectively), when all the trees were *C*Las-positive, the number of fruits was still significantly higher in trees that had been covered with the IPCs for 30 months than in non-covered trees. In the second and third seasons, the average crop yield was between 35 and 55 per 3-tree replicated unit in previously IPC-covered trees compared to only 5 fruits per 3-tree replicated unit in non-IPC trees (Figure 3), with no significant effect of the insecticide rate.

**Figure 4:**
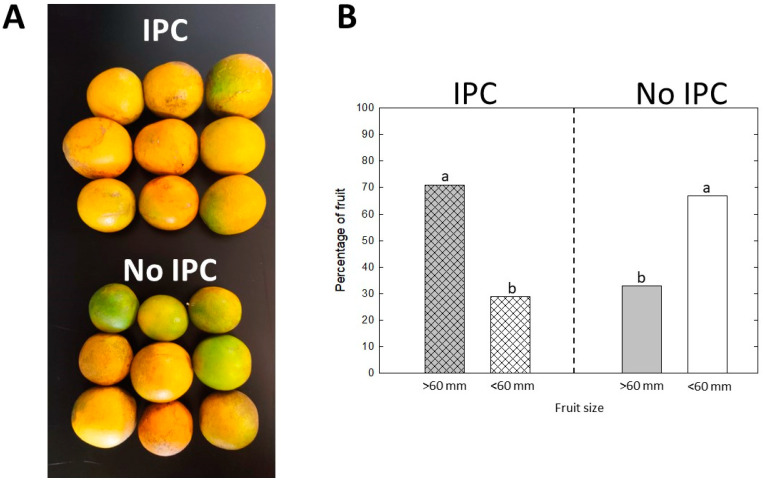
External fruit quality. (**A**) Fruits from IPC-covered trees and non-covered trees. Fruits are from trees 48 months after planting and 18 months after IPC removal (February 2022, second harvesting season). (**B**) Packout of fruit as if for the fresh market (average of second and third seasons). Fruits were divided into larger or smaller than 60 mm size classes. Values are average of two seasons’ data for means of 5 replications of 3 trees ± SD. Different letters indicate significant differences between IPC and non-IPC trees (*p* < 0.05).

Fruit size was significantly affected by IPC in all three seasons. In the second season, when a significant crop was present, we pooled the fruits into two categories, larger or smaller than 60 mm in diameter, which could be compared with fresh fruit classification. In trees originally covered by IPCs, larger fruits were more abundant: 70% of the fruits were larger than 60 mm and 30% were smaller than 60 mm (Figure 4). In non-IPC trees, the reverse was observed: 70% of the fruits were smaller than 60 mm and 30% were larger than 60 mm. Fruit from the third season followed the same pattern, and there was no significant difference between these last two seasons.

We determined the internal and external fruit quality at harvest in all three seasons. Fruits were harvested in mid-February 2021, 2022, and 2023. In trees with IPCs, fruit harvested in 2021 was set while trees were still covered, but completed their last 6 months of development and maturation after IPC removal in August 2021 when the trees started to become infected. Fruit harvested in 2022 and 2023 was set and matured under non-covered conditions in all trees, and all trees were already infected. The results are summarized in Table 2. The Brix values were significantly (*p* < 0.05) higher in fruit from IPC trees compared to non-IPC trees in all three seasons (10.9 vs. 7.6 in season 1, 9.6 vs. 7.0 in season 2, and 7.6 vs. 6.0 in season 3). The Brix/acid ratio was also significantly (*p* < 0.05) higher in IPC trees than non-IPC trees in all three seasons, ranging from 13.6 in season 1 to 8.4 in season 3 in IPC trees compared with 12.5 to 5.4 in non-IPC trees. The external fruit color was better in fruit from IPC trees at harvest in all three seasons, with higher a/b ratios (Table 2).

## 4. Discussion

One major concern for the citrus industry in Florida has been that newly planted trees under endemic HLB conditions, either in solid blocks or as resets, will never produce harvestable fruit, or fruit quality will be poor due to *C*Las infection [2]. Infection greatly reduces fruit quality and exacerbates fruit drop, resulting in growers having to harvest earlier than pre-HLB. The premature harvest time greatly impacts juice quality, as maturation is critical for achieving commercially acceptable juice quality [10]. We have previously shown IPCs’ effectiveness in preventing newly planted trees from becoming infected with *C*Las by excluding the ACP [6]. Uncovering trees after 30 months of IPC protection resulted in a trend of infection similar to that of trees that were not covered at planting. As a result, 100% infection after IPC removal was observed in about 10 months of exposure to ACPs. This reinforces the notion that covering trees with IPCs at planting is currently the best option for growers to keep young trees free from HLB.

Tree growth was promoted by IPCs. Trunk diameter, tree height, and canopy volume were larger than in non-covered trees after 30 months, irrespective of the insecticide dosage [6]. It was not clear if this advantage conferred by IPCs is the result of keeping trees free from HLB, a shading effect, or both combined. Shading can promote tree growth in citrus and improve photosynthesis [14] and may ameliorate HLB symptoms [15]. This possibility merits further evaluation, which is currently being performed in our labs. Some favorable differences in tree growth were maintained for at least 6 months after IPC removal; tree height and LAI were still significantly improved in trees that were covered with IPCs. After IPC removal, insecticide treatment had a significant effect on tree growth, but only for trunk diameter, not tree height. This reinforces that it is critical to continue to protect trees from ACPs once IPCs are removed and trees are left exposed to natural ACP pressures. In any case, the differences observed in tree health parameters between protected and non-protected trees influenced the ability of trees to produce fruit and determined fruit production upon IPC removal.

We monitored fruit set, yield, and quality for three consecutive seasons after IPC removal in both types of trees: trees not covered at the time of planting and, hence, exposed to ACPs (and *C*Las infection) from the beginning of the experiment, and trees protected with IPCs for 30 months and only then exposed to ACPs (and *C*Las infection) for another 30 months after IPC removal. Some varieties, including the most widely planted in Florida for juice production, such as “Hamlin” and “Valencia” sweet oranges, can set fruit under IPCs, without the need for external pollinators. This was the case in the first season in our study when trees bloomed and set fruit while being under IPC covers. After IPC removal in August 2020, during the rapid fruit growth stage due to cell expansion in phase II of fruit development [16], trees were left exposed to psyllids and, therefore, *C*Las infection. However, no fruit drop was observed in this first season even though infection likely occurred during phase II of fruit development. It has been suggested that cell division and/or expansion are impeded in response to HLB-induced changes in hormone homeostasis [17], and that the likeliness of fruit drop before harvest might be determined before fruits reach maturity, and as early as stage I or stage II of fruit development and growth [12]. Fruit yield was consistently higher in trees protected by IPCs in all three seasons. A combination of factors may explain this. First, fruit set was more abundant in all three seasons in trees that were initially covered with IPCs, even though these trees became infected after IPC removal. Second, fruit drop was absent in the first season in IPC-protected trees, and significantly lower than in non-protected trees in the following two seasons, despite *C*Las infection. Fruit drop was around 60% in non-protected trees in all three seasons, which is consistent with observations in commercial groves [18]. Finally, fruit from trees that were initially covered with IPCs was significantly larger than fruit from non-covered trees. It is worth noting that generally, HLB-symptomatic fruit from infected trees is smaller in diameter compared to asymptomatic fruit from infected or from healthy trees [10]. In addition, larger fruits consistently have a higher fruit detachment force (i.e., the force necessary to detach a fruit from the stem through the abscission zone), resulting in less fruit drop [12].

One of the most important effects of HLB is reduced fruit quality. Symptomatic fruits from HLB-affected trees show higher titratable acidity, lower soluble solids, and a lower sugar/acid ratio [10]. This results in enormous economic losses for the industry, as juice quality is negatively affected. In Florida, growers are paid by pound of solids, the weight of the total dissolved sugar solids in each 90 lb (40.82 kg) box of fruit; therefore, juice quality is a very important parameter for the viability of a farm. Peel color is affected by HLB as well. The flavedo in HLB symptomatic fruit tends to be greener than the flavedo from healthy fruit, as color development may only “break” on the stem end, leaving most of the fruit surface green [18].

We monitored the internal and external fruit quality for the three consecutive seasons. Consistently, fruit quality was improved in IPC-covered trees, although eventually, trees became infected after IPC removal. Brix and the sugar-to-acid ratio were significantly higher in fruit from IPC trees in all three seasons. In the first season’s harvest, at the time the trees previously covered with IPCs were exposed to psyllid infestation for 6 months and most of the trees (around 60%) were infected, Brix in the fruit from these trees was significantly higher at 10.9 compared to 7.6 in fruit from the control trees, which were exposed to HLB infection for 36 months. Significant differences were maintained in the subsequent two seasons, although a clear decline in Brix and the sugar-to-acid ratio was observed with each season. This was expected, as the health of the trees was declining with infection, despite the continued insecticide treatments after IPC removal. However, it is worth noting that despite the decline in internal quality, IPCs provided a kick-start to trees resulting in better attributes, such as healthier tree development and the retention of fruit quality under HLB conditions. We also consistently observed better coloration in the flavedo from fruit in IPC-covered trees in all three seasons. “Valencia” is a late-maturation variety, and the fruit color was not fully developed at the time of harvest in early February. In Florida, under HLB-free conditions, “Valencia” fruits reach their full color and internal quality by April or May, but under HLB, it is nearly impossible that trees bear fruit that late in the season, mainly due to the extensive pre-harvest fruit drop. This was the main reason for harvesting fruit in our experiments in February. It is not clear that the color we measured was the result of advanced degreening or less regreening, both due to healthier tree conditions. In any case, IPC protection during the first 30 months after planting imparted favorable conditions that improved the color of the flavedo.

In conclusion, our data support previous reports that IPCs improve tree health under HLB-endemic conditions and show that this tool kick-started the newly planted citrus trees, resulting in higher yields and fruits with better internal and external quality. Based on the results from this study, the benefits from IPC protection may last for at least three consecutive seasons once trees enter the productive age, despite *C*Las-infection within 12 months after IPC removal. Sustained tree health is a goal after IPC removal so that quality fruit production can be attained over time. Additional management practices should be considered after IPC removal to prolong its positive effects. Currently, we are investigating the use of foliar sprays of homobrassinolides, which appear to be promising for retaining health via the induction of SAR immunity and improving young tree vigor [19,20,21]. Research has also shown that oxytetracycline (OTC) administered by trunk injection can effectively reduce fruit drop, increase fruit yield and fruit size, and improve juice quality in young and mature HLB-affected trees [22,23]. We envision a novel Integrated Pest Management (IPM) strategy that would employ IPCs for young tree protection followed by treatment with homobrassinolide or other plant growth regulators to improve growth further and delay *C*Las infection or alleviate HLB symptoms as the trees transition to full commercial fruit production (4–5-year-old trees). This could help to alleviate eventual resistance to insecticides [24]. Insecticide applications alone are not sufficient for *D. citri* management, and pest management tools that can be used to reduce the frequency of pesticide treatment are needed. In any case, IPCs remain the most efficient tool for young tree protection against HLB in endemic conditions [25]. Once trees become infected, the injection of OTC or other antibacterials/immunity inducers would prevent tree decline and ensure the continued production of high-quality fruit. Studies are already in progress to assess these strategies and determine their economic benefits.

## Figures and Tables

**Table 1:** Tree height, canopy volume, leaf area index, scion, and rootstock trunk diameter 6 months after IPC removal.

	6 Months after IPC Removal					30 Months after IPC Removal				
Factor	RS Diameter (mm)	SC Diameter (mm)	Tree Height (m)	Canopy Volume (m^3^)	LAI	RS Diameter (mm)	SC Diameter (mm)	Tree Height (m)	Canopy Volume (m3)	LAI
Cover										
No IPC	55.4a	49.1b	1.6b	0.8a	1.10b	65.82a	58.36b	1.8b	0.99b	1.6b
IPC	59.8a	56.5a	2.1a	0.9a	2.21a	70.17a	67.13a	2.55a	1.4a	3.7a
*p*-value	0.176	0.05 *	<0.001 ***	0.479	0.01 **	0.26	0.001 ***	0.01 **	0.01 **	0.001 ***
Insecticide rate										
Full	61.6a	57.3a	1.9a	1.2a	1.57a	71.4a	61.37a	2.35a	1.54a	2.44a
Half	61.4a	56.8a	1.9a	0.9ab	1.42a	62b	58.36a	2.25a	1.24a	1.97a
No	50.4b	45.2b	1.8a	0.5b	1.97a	51.4c	46.33b	2.1a	0.55b	2.21a
*p*-value	0.012 *	0.018 *	0.534	0.004 **	0.41	0.008 **	0.011 *	0.13	0.01 *	0.41

Different letters within columns indicate significant differences according to Tukey’s HSD test. *, **, and *** denote *p*-values significant at 5%, 1%, and less than 0.1%. SC = scion; RS = rootstock; LAI = leaf area index.

**Table 2:** Summary of external and internal fruit quality. Juice quality and fruit color over three production seasons.

	2020–2021 Season				2021–2022 Season				2022–2023 Season			
	6 Months after IPC Removal				18 Months after IPC Removal				30 Months after IPC Removal			
Factor	Brix	TTA (%)	Ratio	Color a/b	Brix	TTA (%)	Ratio	Color a/b	Brix	TTA (%)	Ratio	Color a/b
No IPC	7.5b	0.6	12.5b	−0.1b	7.0b	1.1b	6.4b	−0.12b	6.4b	1.1b	5.8b	−0.14b
IPC	10.9a	0.8	13.6a	0.2a	9.6a	0.84a	11.4a	0.15a	7.6a	0.9a	8.4a	0.17a
*p*-value	0.009 **	0.29	0.015 *	0.001 **	0.01 *	0.1	0.0004 ***	0.001 **	0.01 *	0.1	0.0001 ***	0.04 *
Insecticide rates												
Full	8.7a	0.8a	10.9a	−0.1	8.2a	0.9a	9.1a	−0.1a	7.0a	1.0a	7a	−0.12a
Half	9.9a	0.8a	12.4a	−0.1	8.3a	0.8a	10.4a	0.05a	6.8a	0.9a	7.5a	0.1a
No	9.2a	0.6a	15.3b	0.15	7.6a	0.9a	8.4a	−0.15a	6.0a	0.9a	6.7a	−0.2a
*p*-value	0.28	0.29	0.015*	0.16	0.23	0.25	0.09	0.08	0.24	0.22	0.14	0.07

Different letters within columns indicate significant differences according to Tukey’s HSD test. *, **, and *** denote *p*-values significant at 5%, 1%, and less than 0.1%. TTA = titratable acidity. Fruits were harvested in February of each year.

## Data Availability

The original contributions presented in the study are included in the article; further inquiries can be directed to the corresponding author.
